# Synchronous Phase-Shifting Interference for High Precision Phase Imaging of Objects Using Common Optics

**DOI:** 10.3390/s23094339

**Published:** 2023-04-27

**Authors:** Jiaxi Zhao, Lin Liu, Tianhe Wang, Xiangzhou Wang, Xiaohui Du, Ruqian Hao, Juanxiu Liu, Jing Zhang

**Affiliations:** School of Optoelectronic Science and Engineering, University of Electronic Science and Technology of China, Chengdu 611731, China

**Keywords:** quantitative phase imaging, synchronous phase-shifting, Mach–Zehnder interferometer, digital holography

## Abstract

Quantitative phase imaging and measurement of surface topography and fluid dynamics for objects, especially for moving objects, is critical in various fields. Although effective, existing synchronous phase-shifting methods may introduce additional phase changes in the light field due to differences in optical paths or need specific optics to implement synchronous phase-shifting, such as the beamsplitter with additional anti-reflective coating and a micro-polarizer array. Therefore, we propose a synchronous phase-shifting method based on the Mach–Zehnder interferometer to tackle these issues in existing methods. The proposed method uses common optics to simultaneously acquire four phase-shifted digital holograms with equal optical paths for object and reference waves. Therefore, it can be used to reconstruct the phase distribution of static and dynamic objects with high precision and high resolution. In the experiment, the theoretical resolution of the proposed system was 1.064 µm while the actual resolution could achieve 1.381 µm, which was confirmed by measuring a phase-only resolution chart. Besides, the dynamic phase imaging of a moving standard object was completed to verify the proposed system’s effectiveness. The experimental results show that our proposed method is suitable and promising in dynamic phase imaging and measurement of moving objects using phase-shifting digital holography.

## 1. Introduction

Phase-shifting digital holography (PSDH) as a non-contacting, full-field and highly accurate phase measurement technology, has evolved as an important tool in the world for surface topography measurement [1,2], biomedical imaging [3,4] and industrial defect detection [5,6]. Unlike off-axis digital holography, PSDH could make full utilization of the Spatial-bandwidth Product (SBP) of the charge-coupled device (CCD) camera and thus has high spatial resolution [7]. In addition, the accuracy of phase measurement and noise level in PSDH is better than that in off-axis digital holography due to the introduction of phase shift and the acquisition of multiple holograms [8,9].

According to the recording method of holographic images, PSDH can be classified mainly as two types of implementations: asynchronous phase-shifting and synchronous phase-shifting. In the past, driving piezoelectric ceramics [10] and changing polarization states [11] have been proposed for asynchronous phase-shifting. However, asynchronous phase-shifting methods need to record multiple holograms several times and thus are only suitable for the situations of observing static objects. Besides, it is difficult to guarantee the accuracy of each step of the phase shift, and it is also difficult to guarantee that the relevant physical quantities will not change during the transformation process of the phase shift in a real experiment, thus affecting the accuracy of phase reconstruction [12,13].

In order to improve the acquisition rate of recording multi-frame phase-shifted holograms, synchronous phase-shifting techniques based on multi-CCDs recording [14,15,16], pixel mask [8,17,18], and parallel splitting [19] have been proposed. These techniques do not require mechanical phase-shifting units and can record multiple phase-shifted holograms simultaneously. Synchronous phase-shifting techniques based on pixel mask require dividing the imaging area of a CCD to apply different phase shifts to different regions of the CCD, thus achieving the acquisition of four phase-shifted digital holograms on the same camera. The advantage of this approach is that only one camera is needed, but the drawback is a decrease in the spatial resolution of digital holograms. Among these techniques, holography methods based on multi-CCDs recording offer the capability to capture holograms with the highest possible spatial resolution without dividing the imaging area of a CCD. In the last thirty years, Smythe et al. [14], Koliopoulos et al. [15] and Sivakumar et al. [16] have proposed representative systems for synchronous phase-shifting techniques based on multi-CCDs recording. Although these systems could be used for reflective samples, they did not ensure that object and reference waves passed through the same optical elements after recombining in the beamsplitter. Therefore, they may result in additional phase changes in the light field. Avner et al. [20] used a polarization-based Linnik interferometer to present a real-time phase shift interference microscopy system. This system could record three phase-shifted holograms simultaneously and ensure the same optical paths for light reaching different CCDs. Zhang et al. [21] proposed a system to rapidly reconstruct the phases of transmissible samples, which could record four phase-shifted holograms simultaneously. However, one of the beamsplitters needed additional anti-reflective coating or reflection-enhancing coating to avoid half-wave loss although this system worked effectively. N.-I. Toto Arellano et al. [22] coupled a rectangular Sagnac interferometer and two Michelson interferometers together to generate four phase-shifted holograms simultaneously. Their system needed a polarizer array before the camera to obtain phase shifts. The function of the polarizer array was to generate varying phase shifts for different regions of a CCD. Siqi Wang et al. [23] proposed a common-path and synchronous phase-shifting of lateral shearing interferometry which also needed a micro-polarizer array for phase shifting. These additional optical elements require specific design, which is difficult to obtain.

In summary, the current synchronous phase-shift methods face challenges in ensuring an equal optical path for both object and reference waves or may require specialized optics to mitigate issues such as half-wave loss and achieve synchronous phase-shift. To address the aforementioned issues, we present a novel synchronous phase-shifting method based on common optics that is very simple and easy to implement. The common optics include beamsplitters, polarizers and wave plates, which are easily obtainable and do not require special processing. Additionally, no additional anti-reflective coating or reflection-enhancing coating is required for each optical element of the system to avoid half-wave loss. Furthermore, both object and reference waves pass through the same optical elements exactly, so that the additional phase changes in the light field can be avoided. This system can be used not only to accurately reconstruct the phase distribution of static objects, but also to efficiently retrieve the phase distribution of dynamic objects with four CCDs recording phase-shifted holograms simultaneously.

## 2. Materials and Methods

In PSDH, the reference wave is imported phase delays to realize phase shifting for each hologram. In this work, we utilized commonly used polarizers and wave plates to introduce additional phases of 0, $\frac{\pi }{2}$, π and $\frac{3\pi }{2}$ in the reference wave respectively, and recorded four holograms simultaneously. Specifically, the intensity of each phase-shifted hologram $I_{i}(x,y)$ is given by:

(1) $$I_{i}(x,y)=I_{0}(x,y)+2A_{O}(x,y)A_{R}(x,y)*\cos[\Delta \varphi (x,y)-\delta_{i}],$$

where (x,y) are the coordinates on the detector plane, $I_{0}(x,y)$ is the intensity of background (DC level of the hologram), $A_{O}(x,y)$ and $A_{R}(x,y)$ are the amplitude of object and reference waves respectively, Δφ(x,y) is the phase difference between object wave and reference wave, the phase delay $\delta_{i}$ is defined as follows:

(2) $$\delta_{i}=i*\frac{\pi }{2}(i=0,1,2,3),$$

According to these phase-shifted holograms, $I_{0}(x,y)$ can be eliminated by some algebraic operations and Δφ(x,y) is derived as follows:

(3) $$\Delta \varphi (x,y)=\arctan[\frac{I_{1}(x,y)-I_{3}(x,y)}{I_{0}(x,y)-I_{2}(x,y)}].$$

**Figure 1:**
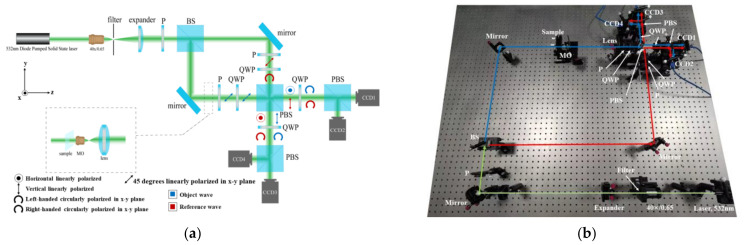
The designed PSDH system. (**a**) The schematic of the designed PSDH system; (**b**) The physical diagram of the designed system. P: linear polarization; BS: non-polarizing beamsplitter cube; QWP: quarter-wave plate; PBS: polarizing beamsplitter cube; MO: microscope objective.

Our system based on the Mach–Zehnder interferometer is illustrated in Figure 1. The light source is a diode pumped solid state laser (Edmund, a maximum output power of 10 mW @ λ = 532 nm). After being filtered and expanded, a coherent beam emerging from the source is incident on a 50/50 non-polarizing beamsplitter cube. The coherent beam is then split into object and reference paths. The reference wave continues to travel through a linear polarizer and a quarter-wave plate sequentially to produce circularly polarized light. The linear polarizer is oriented at 45 degrees relative to the x and y basis vectors of the system while the quarter-wave plate is oriented parallel with the basis vectors.

The object wave travels through the observed sample and is introduced in a phase delay of the sample. Then it continues to travel through a linear polarizer, and a quarter-wave plate sequentially to produce polarized light. Because its axis is parallel with that of the polarizer, the quarter-wave plate on the object path does not change the polarization state of the object wave. It should be noted that we used the quarter-wave plate to keep the influence of these optical elements on object and reference waves consistent. The object and reference waves will recombine at the 50/50 polarizing beamsplitter cube. Afterward, their quadrature components are separated by reflection and transmission and will travel in vertical directions. The remixable waves outputting from the polarizing beamsplitter cube travel through a quarter-wave plate oriented at 45 degrees, and their polarization changes from the linear polarization state to the circular polarization state. Then the quadrature components of the mixed circularly polarized light are separated by a 50/50 polarizing beamsplitter cube. Finally, each quadrature component is recorded as a phase-shifted hologram by a CCD.

In order to further explain the principle of the synchronous phase-shift method realized in our system, we provided a concrete mathematical description. The system assumed that the coherent beam from the laser is an ideal planar light field after being filtered and expanded. Besides, the thermal noise of the laser, aberration of the optics, shot noise of these CCDs and energy loss of the light are ignored in the following mathematical derivation. The planar coherent beam travels through the polarizer to produce the linearly polarized light. The electric field of a light wave in an arbitrary polarization state can be expressed using a vector notation as:

(4) $$E=E_{x}x+E_{y}y=\left(\frac{E_{x}}{E_{y}}\right),$$

where E is the light wave, x and y are the basis vectors of the plane perpendicular to the optic axis, which are parallel to the vibrating directions of p-polarized light and s-polarized light respectively; $E_{x}$ and $E_{y}$ are the x and y components for the complex amplitudes of the electric field. After traveling through the linear polarization, the light wave is linearly polarized. The linearly polarized light then travels through the 50/50 non-polarizing beamsplitter cube, which splits the beam into object and reference paths. After traveling through the observed sample, the object beam carries the information of the sample. Then both the object beam and the reference beam travel through a linear polarizer oriented at 45 degrees to x and are set to a linear polarization state. Supposing the complex amplitude of the object wave is $E_{O}$ and the complex amplitude of the reference wave is $E_{R}$, the object wave and the reference wave that travel through the linear polarizer can be written as:

(5) $$\left\{\begin{array}{l} E_{O,P}=\left[\begin{array}{cc} 1 & 1\\ 1 & 1 \end{array}\right]E_{O}=A_{O}e^{j\varphi_{o}}(x+y)\\ E_{R,P}=\left[\begin{array}{cc} 1 & 1\\ 1 & 1 \end{array}\right]E_{R}=A_{R}e^{j\varphi_{r}}(x+y) \end{array}\right.,$$

where $E_{O,P}$ and $E_{R,P}$ are the object wave and the reference wave that travel through the linear polarizer respectively, $\left[\begin{array}{cc} 1 & 1\\ 1 & 1 \end{array}\right]$ is the Jones matrix for the linear polarizer oriented at 45 degrees to x, $A_{O}$ and $A_{R}$ are the amplitude of the object and reference waves respectively, which are theoretically equal; $\varphi_{o}$ and $\varphi_{r}$ are the phase of the object and reference waves respectively, j is an imaginary unit. After traveling through the 45-degree quarter-wave plate, the 45-degree polarized object wave keeps its polarization invariable while travelling through the observed sample. Then $E_{O,P}$ becomes as:

(6) $$E_{O,P,}{}_{\frac{1}{4}}=\left[\begin{array}{cc} 1 & -i\\ -i & 1 \end{array}\right]E_{O,P}=\alpha A_{O}e^{j(\varphi_{o}+\phi )}(x+y),$$

where $E_{O,P,}{}_{\frac{1}{4}}$ is the output light wave after $E_{O,P}$ traveling through the quarter-wave plate, $\left[\begin{array}{cc} 1 & -i\\ -i & 1 \end{array}\right]$ is the Jones matrix for the quarter-wave plate with its fast axis oriented at 45 degrees to x, α and ϕ are the amplitude and the phase changes introduced by the sample, respectively. The 45-degree polarized reference wave travels through the quarter-wave plate with its fast axis parallel to x, producing circularly polarized light. Then $E_{R,P}$ becomes as:

(7) $$E_{R,P,}{}_{\frac{1}{4}}=\left[\begin{array}{cc} 1 & 0\\ 0 & i \end{array}\right]E_{R,P}=A_{R}e^{j\varphi_{r}}(x+jy),$$

where $E_{R,P,}{}_{\frac{1}{4}}$ is the output light wave after $E_{R,P}$ traveling through the quarter-wave plate, $\left[\begin{array}{cc} 1 & 0\\ 0 & i \end{array}\right]$ is the Jones matrix for the quarter-wave plate with its fast axis parallel to x. The object and reference waves recombine in the 50/50 polarizing beamsplitter cube. Their orthogonal components are separated and output as transmission and reflection respectively:

(8) $$\left\{\begin{array}{l} E_{O}{}_{//}=\frac{1}{\sqrt{2}}\left[\begin{array}{cc} 1 & 0\\ 0 & 0 \end{array}\right]E_{O,P,}{}_{\frac{1}{4}}=\frac{1}{\sqrt{2}}\alpha A_{O}e^{j\varphi_{o}}x\\ E_{O}{}_{⊥}=\frac{1}{\sqrt{2}}\left[\begin{array}{cc} 0 & 0\\ 0 & -1 \end{array}\right]E_{O,P,}{}_{\frac{1}{4}}=-\frac{1}{\sqrt{2}}\alpha A_{O}e^{j\varphi_{o}}y\\ E_{R}{}_{//}=\frac{1}{\sqrt{2}}\left[\begin{array}{cc} 1 & 0\\ 0 & 0 \end{array}\right]E_{R,P,}{}_{\frac{1}{4}}=\frac{1}{\sqrt{2}}A_{R}e^{j\varphi_{r}}x\\ E_{R}{}_{⊥}=\frac{1}{\sqrt{2}}\left[\begin{array}{cc} 0 & 0\\ 0 & -1 \end{array}\right]E_{R,P,}{}_{\frac{1}{4}}=-\frac{1}{\sqrt{2}}A_{R}e^{j\varphi_{r}}jy \end{array}\right.,$$

where $E_{O}{}_{//}$ and $E_{R}{}_{//}$ are parallel to the vibrating direction of p-polarized light, $E_{O}{}_{⊥}$ and $E_{R}{}_{⊥}$ are parallel to the vibrating direction of s-polarized light. $\left[\begin{array}{cc} 1 & 0\\ 0 & 0 \end{array}\right]$ is the Jones matrix for transmission of the polarization beamsplitter cube, $\left[\begin{array}{cc} 0 & 0\\ 0 & -1 \end{array}\right]$ is the Jones matrix for reflection of the polarization beamsplitter cube considering the existence of half-wave loss. The two sets of mixed orthogonal components output $M_{0}$ can $M_{1}$ be expressed as:

(9) $$\left\{\begin{array}{l} M_{0}=E_{O}{}_{//}+E_{R}{}_{⊥}=\frac{1}{\sqrt{2}}\alpha A_{O}e^{j\varphi_{o}}x-\frac{1}{\sqrt{2}}A_{R}e^{j\varphi_{r}}jy\\ M_{1}=E_{O}{}_{⊥}+E_{R}{}_{//}=-\frac{1}{\sqrt{2}}\alpha A_{O}e^{j\varphi_{o}}y+\frac{1}{\sqrt{2}}A_{R}e^{j\varphi_{r}}x \end{array}\right.,$$

The mixed orthogonal components pass through a quarter-wave plate oriented at 45 degrees relative to the x and y basis vectors respectively to produce mixed circularly polarized light. Each of the circularly polarized light fields is:

(10) $$\left\{\begin{array}{l} E_{O}{{}_{//,}}_{\frac{1}{4}}=\left[\begin{array}{cc} 1 & i\\ i & 1 \end{array}\right]E_{O}{}_{//}=\frac{1}{\sqrt{2}}\alpha A_{O}e^{j\varphi_{o}}(x+jy)\\ E_{O}{{}_{⊥}}_{,\frac{1}{4}}=\left[\begin{array}{cc} 1 & i\\ i & 1 \end{array}\right]E_{O}{}_{⊥}=\frac{1}{\sqrt{2}}\alpha A_{O}e^{j\varphi_{o}}(-jx-y)\\ E_{R}{{}_{//}}_{,\frac{1}{4}}=\left[\begin{array}{cc} 1 & i\\ i & 1 \end{array}\right]E_{R}{}_{//}=\frac{1}{\sqrt{2}}A_{R}e^{j\varphi_{r}}(x+jy)\\ E_{R}{{}_{⊥}}_{,\frac{1}{4}}=\left[\begin{array}{cc} 1 & i\\ i & 1 \end{array}\right]E_{R}{}_{⊥}=\frac{1}{\sqrt{2}}A_{R}e^{j\varphi_{r}}(x-jy) \end{array}\right.,$$

where $E_{O}{{}_{//,}}_{\frac{1}{4}}$, $E_{O}{{}_{⊥}}_{,\frac{1}{4}}$, $E_{R}{{}_{//}}_{,\frac{1}{4}}$, and $E_{R}{{}_{⊥}}_{,\frac{1}{4}}$ are the optical field distributions of the components ($E_{O}{}_{//}$, $E_{O}{}_{⊥}$, $E_{R}{}_{//}$ and $E_{R}{}_{⊥}$) passing through the 45-degree quarter-wave plate. $\left[\begin{array}{cc} 1 & i\\ i & 1 \end{array}\right]$ is the Jones matrix for the quarter-wave plate with its slow axis oriented at 45 degrees to x. $M_{0}$ and $M_{1}$ both contain two polarization states of light fields. The last 50/50 polarizing beamsplitter cube separates the orthogonal components of the mixed circularly polarized light and finally is recorded by four CCDs. The distribution of light intensity on each CCD can be expressed as:

(11) $$\left\{\begin{array}{l} I_{CCD1}={\left\|\frac{1}{\sqrt{2}}\left[\begin{array}{cc} 1 & 0\\ 0 & 0 \end{array}\right](E_{O}{{}_{//,}}_{\frac{1}{4}}+E_{R}{{}_{⊥}}_{,\frac{1}{4}})\right\|}^{2}={\left\|\frac{1}{2}(\alpha A_{O}e^{j\varphi_{o}}x+A_{R}e^{j\varphi_{r}}x)\right\|}^{2}\\ I_{CCD2}={\left\|\frac{1}{\sqrt{2}}\left[\begin{array}{cc} 0 & 0\\ 0 & -1 \end{array}\right](E_{O}{{}_{//,}}_{\frac{1}{4}}+E_{R}{{}_{⊥}}_{,\frac{1}{4}})\right\|}^{2}={\left\|\frac{1}{2}(-\alpha A_{O}e^{j\varphi_{o}}jy+A_{R}e^{j\varphi_{r}}jy)\right\|}^{2}\\ I_{CCD3}={\left\|\frac{1}{\sqrt{2}}\left[\begin{array}{cc} 1 & 0\\ 0 & 0 \end{array}\right](E_{O}{{}_{⊥}}_{,\frac{1}{4}}+E_{R}{{}_{//}}_{,\frac{1}{4}})\right\|}^{2}={\left\|\frac{1}{2}(-\alpha A_{O}e^{j\varphi_{o}}jx+A_{R}e^{j\varphi_{r}}x)\right\|}^{2}\\ I_{CCD4}={\left\|\frac{1}{\sqrt{2}}\left[\begin{array}{cc} 0 & 0\\ 0 & -1 \end{array}\right](E_{O}{{}_{⊥}}_{,\frac{1}{4}}+E_{R}{{}_{//}}_{,\frac{1}{4}})\right\|}^{2}={\left\|\frac{1}{2}(\alpha A_{O}e^{j\varphi_{o}}y-A_{R}e^{j\varphi_{r}}jy)\right\|}^{2} \end{array}\right.,$$

where ${\left\|\right\|}^{2}$ denotes modulo operation of complex amplitude, then Equation (11) can be rewritten as:

(12) $$\left\{\begin{array}{l} I_{CCD1}=\frac{1}{4}(\alpha^{2}A_{O}{}^{2}+A_{R}{}^{2}+\alpha A_{O}A_{R}e^{j\Delta \varphi }+\alpha A_{O}A_{R}e^{-j\Delta \varphi })\\ I_{CCD2}=\frac{1}{4}(\alpha^{2}A_{O}{}^{2}+A_{R}{}^{2}-\alpha A_{O}A_{R}e^{j\Delta \varphi }-\alpha A_{O}A_{R}e^{-j\Delta \varphi })\\ I_{CCD3}=\frac{1}{4}(\alpha^{2}A_{O}{}^{2}+A_{R}{}^{2}-j\alpha A_{O}A_{R}e^{j\Delta \varphi }+j\alpha A_{O}A_{R}e^{-j\Delta \varphi })\\ I_{CCD4}=\frac{1}{4}(\alpha^{2}A_{O}{}^{2}+A_{R}{}^{2}+j\alpha A_{O}A_{R}e^{j\Delta \varphi }-j\alpha A_{O}A_{R}e^{-j\Delta \varphi }) \end{array}\right.,$$

where $\Delta \varphi =\varphi_{o}-\varphi_{r}$. According to Euler’s formula, Equation (12) can be simplified as:

(13) $$\left\{\begin{array}{l} I_{CCD1}=\frac{1}{4}[\alpha^{2}A_{O}{}^{2}+A_{R}{}^{2}+2\alpha A_{O}A_{R}\cos(\Delta \varphi )]\\ I_{CCD2}=\frac{1}{4}[\alpha^{2}A_{O}{}^{2}+A_{R}{}^{2}-2\alpha A_{O}A_{R}\cos(\Delta \varphi )]\\ I_{CCD3}=\frac{1}{4}[\alpha^{2}A_{O}{}^{2}+A_{R}{}^{2}+2\alpha A_{O}A_{R}\sin(\Delta \varphi )]\\ I_{CCD4}=\frac{1}{4}[\alpha^{2}A_{O}{}^{2}+A_{R}{}^{2}-2\alpha A_{O}A_{R}\sin(\Delta \varphi )] \end{array}\right.,$$

where the quadratic component denotes the superposition of direct components of the object and reference waves, the third term denotes the modulation of light intensity by the interference between the object and reference waves. After eliminating the direct component, the phase delay Δφ of the sample can be expressed as:

(14) $$\Delta \varphi =\arctan[\frac{I_{CCD3}-I_{CCD4}}{I_{CCD1}-I_{CCD2}}],$$

If the refractive index of the sample is equally distributed and known, the height distribution of its surface can be recovered further [24]:

(15) $$\Delta h=\frac{\lambda *\Delta \varphi }{2\pi (n_{s}-n_{m})}.$$

where Δh the thickness or height map of the sample, λ is the wavelength of the light source, $n_{s}$ and $n_{m}$ are the refractive index of the sample and the surrounding medium respectively.

## 3. Results

In this section, we described the performance of the system for measuring quantitative phase information of the undyed transparent samples. To thoroughly evaluate the effectiveness of our proposed method, we chose the phase-only resolution chart as the static phase object and polymethyl methacrylate (PMMA) pellets as moving objects to reconstruct their phases.

### 3.1. Working Flow of Reconstruction Phase Distribution

**Figure 2:**
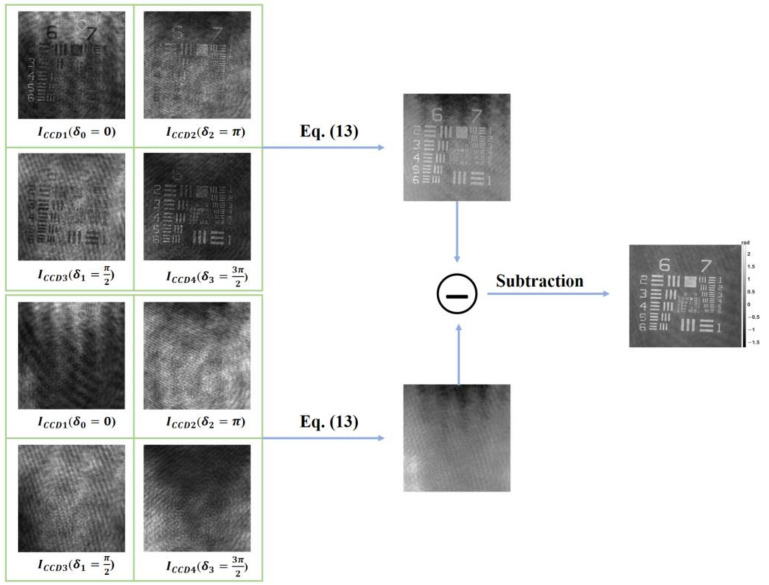
The workflow of reconstructing phase using the proposed system. The first column displays four phase-shifted images (including the holograms of the sample and the calibration holograms). The second column are the reconstructed phases. The last column illustrates compensated phase distribution of the resolution chart.

In this section, we used a phase-only USAF 1951 resolution chart (Quantitative Phase Microscopy Target, Benchmark Technologies, Boston, MA, USA) as the observed sample to introduce the process of reconstructing the phase. Figure 2 shows the workflow of the proposed system. In order to align the four holograms, an intensity-based registration algorithm [25] was used for the pretreatment. The goal of the intensity-based registration algorithm is to find a geometric transformation. It utilizes the pixels of images to construct a feature space, where the statistical information is used to calculate the similarity between the moving image that we want to register and the fixed image (target image). By continuously computing the similarity between the moving image and the fixed image, the algorithm seeks to find the geometric transformation that maximizes the similarity. By employing the geometric transformation, the moving image can be accurately aligned with the fixed image, ensuring a one-to-one correspondence between points that occupy the same spatial positions in both images. It should be noted that the system only needs registration once to calculate the geometric transformation of the four holograms. Once the holograms are aligned, the phase distribution of the sample can be reconstructed according to Equation (14). The height distribution can be recovered based on the phase distribution according to Equation (15). Furthermore, the calibration phase (without the sample) needs to be measured and subtracted from the phase distribution of the samples to compensate for the phase aberration introduced by the MO and the system.

### 3.2. Analysis of Resolution

**Figure 3:**
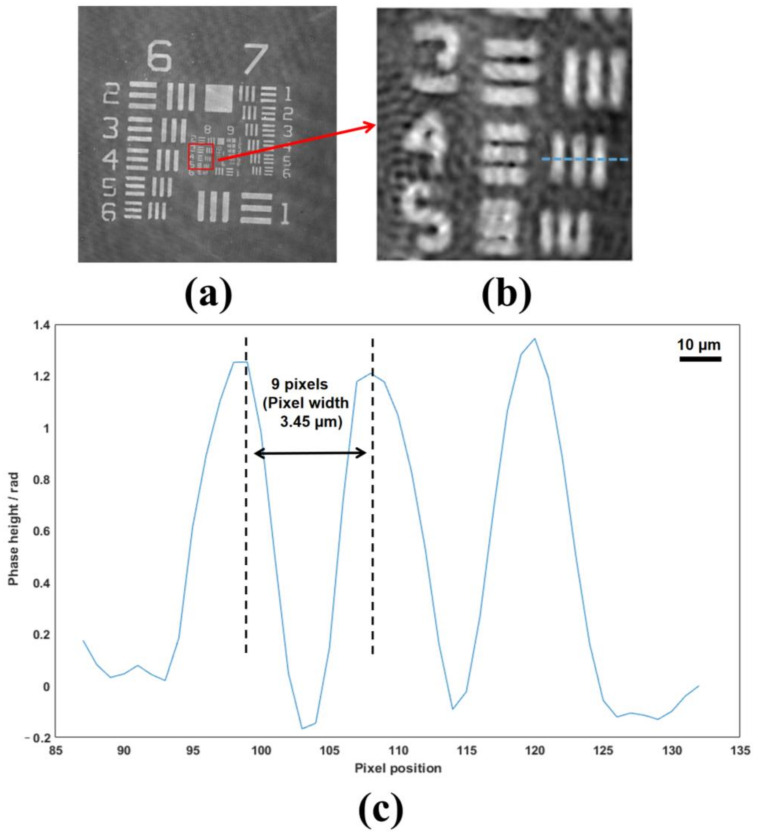
Evaluation of the resolution. (**a**) The reconstructed phase of the resolution chart; (**b**) The enlargement of the red square; (**c**) The phase heights across the designated lines in (**b**).

The phase-only USAF 1951 resolution chart was used for confirming the resolution of our system. The phase elements of the resolution chart were magnified by a microscope objective (Olympus PLN 10×, NA = 0.25, FL = 18 mm). An achromatic doublet (f = 250 mm, D = 50.8 mm) was used to collect the light output from the objective and transmits the clear image to the CCD (DAHENG, MER2-502-79U3CL, 2448 × 2048 @ 79 fps). The pixel pitch of each CCD used by the system is 3.45 µm and the holograms were recorded for reconstructing the phase on the basis of the workflow shown in Figure 2. According to the numerical aperture of the system and the wavelength of the coherent light source [26]:

(16) $$\gamma =\frac{\lambda }{2NA}.$$

where γ is the theoretical resolution, NA is the numerical aperture of the microscope objective. According to Equation (16), the transverse resolution of our system can achieve 1.064 µm in theory. However, the reconstruction accuracy of object images can be affected by various factors, such as the adjustment precision of the optical system, the extinction ratio of the polarizer array, random noise of the camera, interpolation errors, and spatial non-uniformity of the laser. Therefore, the actual resolution is generally lower than the theoretical value. In the proposed system, due to both the object and reference waves passing through the same optical elements precisely, the effects of the external environment on the object wave and reference wave can be compensated, including factors such as the adjustment precision of the optical system, the extinction ratio of the polarizer array, and spatial nonuniformity of the laser. In addition, during phase imaging of objects, measuring and subtracting the calibration phase from the phase distribution of the objects is necessary to compensate for phase aberration. This process significantly reduces the impact of random noise from the camera and interpolation errors on the reconstruction accuracy. Figure 3 shows the result of the resolution evaluation, which indicates that our reconstruction can distinguish element 4 of group 8 and corresponds to a line width of 1.381 µm on the resolution chart [27]. The actual resolution was 0.317 µm higher than the theoretical resolution, which showed that the measurement accuracy was higher than that of [11] and completely acceptable in the actual measurement environment.

### 3.3. Static Phase Measurement for Thin Object

**Figure 4:**
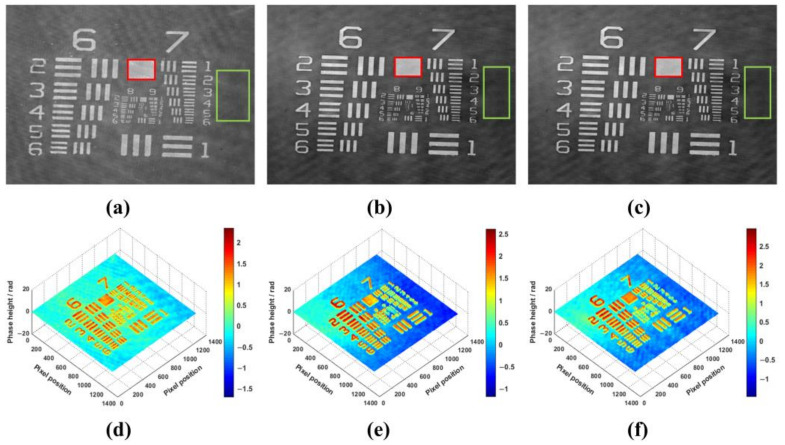
Phase reconstruction of a static phase object. (**a**) Reconstructed phase of 200 nm; (**b**) Reconstructed phase of 300 nm; (**c**) Reconstructed phase of 350 nm; (**d**) The 3D map of (**a**); (**e**) The 3D map of (**b**); (**f**) The 3D map of (**c**).

**Figure 5:**
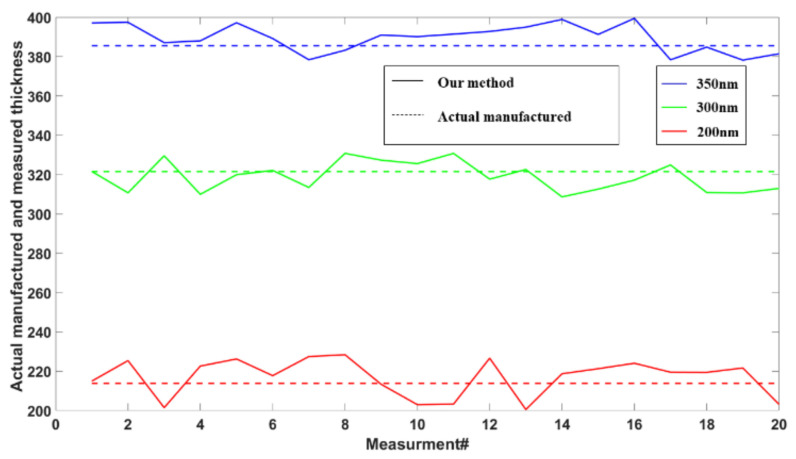
Thickness of the resolution chart reconstructed by the proposed system.

We used the phase-only USAF 1951 resolution chart as a static phase object to reconstruct its phase and corresponding thickness. The various elements of the resolution chart are etched onto the surface of the glass substrate, with a refractive index of 1.52 for both the substrate and the elements. When light passes through the resolution chart, the substrate and elements introduce different phase delays into the optical field. By evaluating the phase difference between the square elements (dashed red squares) and the substrate (dashed green rectangle), the height difference between the elements of the resolution chart and the substrate can be calculated. We first measured the phases of the 200 nm to 350 nm elements on the resolution chart. The reconstructed phases of 200 nm, 300 nm, and 350 nm using our system were presented in Figure 4. In order to observe the reconstruction results more intuitively, their three-dimensional (3D) maps were also shown in Figure 4d–f. As can be seen from Figure 4a–f, the proposed system successfully reconstructed the phases of the 200 nm to 350 nm elements on the resolution chart. According to the known refractive index, we could calculate the thickness distribution of the resolution chart. However, due to the errors in the manufacturing process, the actual manufactured thicknesses have some unavoidable differences. In order to make the calculation results more convincing, the thickness for each chart was estimated 20 times and plotted in Figure 5. Moreover, the average values of the thicknesses were taken as the results of measurement to compare with the actual manufactured thicknesses of the resolution chart in Table 1. As indicated in Table 1, the maximum difference of the thickness between the proposed method and actual manufactured thickness was 4 nm while the minimum difference of the thickness was just 2.6 nm. The above experimental results indicated that the proposed system has a good performance in reconstructing the phase and thickness of a static phase object.

### 3.4. Dynamic Phase Measurement of a Standard Object

**Figure 6:**
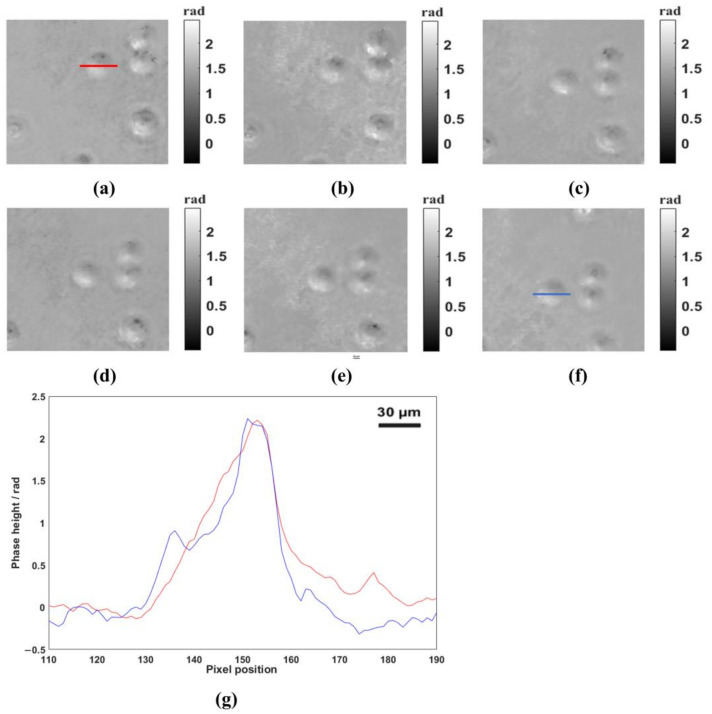
Dynamic phase measurement of the PMMA pellets. (**a**–**f**) are the phase maps reconstructed at 0.5 s intervals over a 3-s period. (**g**) The phase heights across the red line in (**a**) and blue line in (**f**).

**Figure 7:**
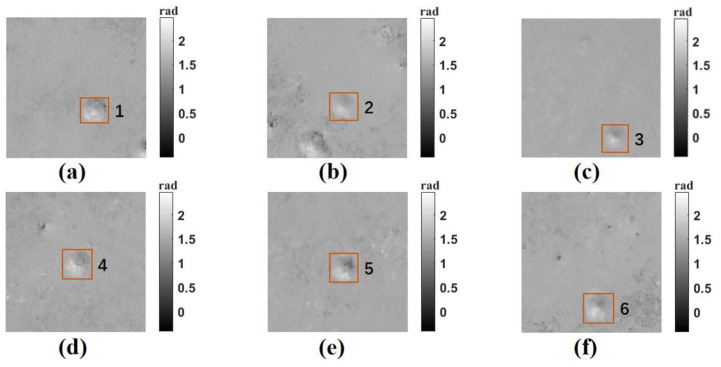
Dynamic phase image of the new PMMA pellets. (**a**–**f**) are the phase maps reconstructed of the new PMMA pellets.

To verify the ability to reconstruct dynamic objects of our system, four imaging cameras were used to record a standard object at approximately 30 frames per second. As a standard object, its attribute parameters are strictly consistent with the manufacturer’s specifications, with an error of no more than 5%, and it has a high degree of recognition among peers. So, we chose the PMMA pellets (refractive index of 1.49) with a diameter of 10 µm as the standard object. We added PMMA pellets to mirror oil (refractive index of 1.51) and used the aforementioned microscopic objective and lens to image. Because PMMA pellets were placed vertically, they will move downward under the influence of gravity. The photography and the recording schematic were displayed in Figure 6. Figure 6a–e are phase images reconstructed from holograms recorded by the four cameras. In this study, the minimum norm methods [28] were used to obtain the unwrapped phase image. From Figure 6a–e, we can clearly see the movement state of PMMA pellets from top to bottom, which demonstrated the feasibility of the proposed system to reconstruct the phase distribution of moving objects. The phase distribution of the red line and blue line indicated in Figure 6a,f are presented in Figure 6g. As shown in Figure 6g, the values of the highest points of the red line and the blue line were 2.33 rad and 2.35 rad respectively, and the corresponding thicknesses were 9.87 µm and 9.95 µm respectively. In addition, we also calculated the thickness of the all intact PMMA pellets in Figure 6a–e, and the average thickness is 10.12 µm. In order to make the measured results reliable, we reacquired 20 holographic images of the PMMA pellets and selected six images from the 20 holographic images as the measurement objects. All of the six images contained the PMMA pellets with the clearest phase and boundary. The phase images and corresponding measurement results were shown in Figure 7 and Table 2, respectively. To facilitate the statistical measurement of the results, brown rectangular boxes and numbers were added to each PMMA pellet measurement object in Figure 7 for the purpose of distinguishing different PMMA pellets. As shown in Table 2, the measured average thickness of PMMA pellets was 10.14 µm, which differed from the average diameter of PMMA pellets (10.17 µm) by only 0.03 µm. It should be noted that all PMMA pellets cannot strictly guarantee a uniform diameter of 10 µm and defocus existing in the process of dynamic recording may also cause the measuring errors. These experimental data indicated the accuracy of the proposed system.

## 4. Conclusions

In this study, we established a quantitative phase imaging system that can be implemented using simple optical components without the need for special processes. Besides, the system could ensure that both the object and reference waves traverse through the same optical elements, thereby avoiding any additional alterations in the optical field. When capturing the phase-shifted hologram, the proposed system is capable of recording four phase-shifted holograms simultaneously. By reconstructing the phase of the resolution chart, we confirmed the high resolution of the system and successfully reconstructed the phase distribution of thin samples. Subsequently, we successfully recorded and reconstructed the dynamic phase distribution of PMMA pellets, demonstrating the ability of the system to measure moving objects. The above two experiments have demonstrated that our system is capable of accurately measuring the phases of both static and dynamic objects without sacrificing resolution. It is expected that this proposed system will contribute to the field of surface topography measurement and fluid dynamics. As a fast, non-destructive, and high-resolution measurement method, synchronous phase-shifting digital holography has the ability to study cellular dynamics and morphological changes [29,30]. The proposed system can also quantitatively investigate biological specimens such as dynamic living cells. Furthermore, our system can be transformed into a reflective synchronous phase-shift interferometer for the semiconductor industry, such as rapidly inspecting wafer defects and flatness after appropriate modifications such as [20].

Note that the proposed system still has some limitations. Although our system can be easily implemented with common optics, it may result in a bulky optical system and higher costs due to the complexity of the components required. In order to accurately reconstruct the phase image of the object, our proposed method relies on an image registration algorithm that aligns the holographic images captured by the four CCDs. This alignment process is a crucial step that ensures precise alignment of the images before proceeding with the phase reconstruction, ultimately leading to reliable and accurate results. Therefore, our main focus will be on simplifying the structure of the synchronous phase-shift system and optimizing the image registration algorithm.

## Figures and Tables

**Table 1:** Average thickness of the resolution chart using the proposed method.

Expected Thickness	Actual Manufactured Thickness	The Proposed Method
200 nm	213.8 nm	216.9 nm
300 nm	321.5 nm	318.9 nm
350 nm	385.5 nm	389.5 nm

**Table 2:** Measurement results of the new PMMA pellets in Figure 7.

Number	Diameter (µm)	Phase Height (rad)	Thickness (µm)
1	9.85	2.36	9.98
2	10.24	2.43	10.30
3	9.72	2.33	9.88
4	10.40	2.41	10.22
5	10.38	2.39	10.12
6	10.41	2.44	10.34
Average	10.17	2.39	10.14

## Data Availability

No data were generated or analyzed in the presented research.
